# Pregnancy outcome in women with antiphospholipid syndrome and alloimmunity: a case report

**DOI:** 10.1590/S1516-31802003000600006

**Published:** 2003-11-06

**Authors:** Serguei Abel Castañeda Ospina, Wálter Darío Cardona Maya, Julio César Bueno Sánchez, Ángela Patricia Cadavid Jaramillo

**Keywords:** Antiphospholipid syndrome, Habitual abortion, Heparin, Aspirin, Immunotherapy, Síndrome do anticorpo antifosfolípide, Aborto habitual, Heparina, Aspirina, Imunoterapia

## Abstract

**CONTEXT:**

Patients with antiphospholipid syndrome and alloimmunity have poor pregnancy outcomes. Several diagnostic and therapeutic options exist for these disorders, although there is no consensus as to the best treatment.

**CASE REPORT:**

We present here the clinical course and treatment of a woman with a history of two miscarriages who joined our program 10 years ago and has been followed up ever since. After antiphospholipid syndrome and alloimmune failure were diagnosed, she was given preconceptional treatment using unfractionated heparin, aspirin, prednisone and lymphocyte immunizations. She delivered two premature babies in the following two pregnancies. At present both children are healthy and are attending school. The fifth pregnancy was unsuccessful, in spite of having undergone a similar but postconceptional therapeutic scheme. We discuss this case focusing on the pathogenic mechanisms and the therapeutic aspects of these disorders.

## RESUMO

**CONTEXTO:**

Mulheres com síndrome do anticorpo antifosfolípide e aloimunidade têm um mau prognóstico de gravidez. Existem muitas opções para o diagnóstico e o tratamento destas doenças, embora o tratamento mais adequado não esteja estabelecido.

**RELATO DE CASO:**

Apresentamos a evolução clínica e o tratamento de uma mulher com antecedentes de dois abortos e que vem sendo acompanhada, há 10 anos, em nosso serviço. Após o diagnóstico da síndrome do anticorpo antifosfolípide e de aloimunidade,, a paciente recebeu tratamento com heparina, aspirina e prednisona, além de imunizações com linfócitos. Nas duas gravidezes subseqüentes, teve dois bebês prematuros, que evoluíram com crescimento e desenvolvimento normais. A quinta gravidez não foi bem-sucedida, apesar de a paciente ter recebido um tratamento semelhante ao utilizado previamente embora após o início da gravidez, não planejada. Na discussão deste caso, enfatizamos os mecanismos patogênicos e o tratamento destas doenças.

## INTRODUCTION

Antiphospholipid syndrome is an autoimmune multisystemic disorder diagnosed by the presence of lupus anticoagulant and anticardiolipin antibodies in association with venous and/or arterial thrombosis or pregnancy complications. These criteria were proposed in the Eighth International Symposium on Antiphospholipid Antibodies and by the American Autoimmune Related Diseases Association.^1^ Rai et al. reported that, among the women with antiphospholipid syndrome tested, 90% presented first trimester pregnancy losses compared with 34% of control women.^2^ The pathogenic mechanisms responsible for the failure of implantation and placentation are unknown. However, antiphospholipid syndrome has been associated with events such as thrombosis, vascular injury and vasoconstriction, all of them possible causes of abnormally reduced maternalfetal interface blood flow. In those cases where pregnancies are successful, these patients present a high risk for intrauterine growth restriction, severe pregnancy-induced hypertension, prematurity and abruptio placentae.^3^

On the other hand, a woman with recurrent pregnancy losses may also present alteration of the maternal immune response against paternal alloantigens expressed by the fetus, the so-called alloimmune failure. It has been hypothesized that allogenic recognition of the fetus by the maternal immune system is a crucial process because it induces secretion of cytokines, such as macrophage-granulocytes and monocyte colony stimulating factors, which promote trophoblastic growth and differentiation.^4^

In the present case report, we describe a woman with all the criteria for antiphospholipid syndrome and alloimmune failure, who has been followed up over a 10-year period.

## CASE

A 40-year-old woman had two first trimester pregnancy losses (at 8 and 6 weeks of gestation) without treatment. In addition, she developed deep vein thrombosis one month after her second miscarriage.

Lupus anticoagulant measured by activated partial thromboplastin time was positive and immunoglobulin G (IgG) anticardiolipin antibodies tested by the enzymelinked immunosorbent assay (ELISA) showed values above 80 GPL (IgG phospholipid units), as determined following the supplier's instructions (Quanta Lite™, Inova Diagnostics, San Diego, California). Likewise, positive titers of IgG and IgM antibodies against phosphatidylserine, phosphatidylinositol, phosphatidylglycerol, phosphatidic acid and cardiolipin were found, when a qualitative method, described by Kwak et al., was used.5 Also, IgG anti-beta-2 glycoprotein I antibodies were positive using a commercial ELISA kit (Quanta Lite™, Inova Diagnostics, San Diego, California).

On the other hand, the patient was diagnosed for alloimmune failure, determined by the absence of blocking factors in a mixed lymphocyte reaction. This method described by Kwak et al.^5^ was adapted in our laboratory.^6^

The patient was treated with unfractionated heparin (5,000 units bid, subcutaneous) beginning on day +1 postovulation and until the 34^th^ week. Low doses of aspirin 100 mg/day orally and prednisone 10 mg bid were administered orally, starting on day 1 of the menstrual cycle. Aspirin was stopped two weeks before the probable date of delivery and prednisone was gradually discontinued from week 24 to week 28. Additionally, the patient received two donor lymphocyte immunizations within an eight-week interval before pregnancy and a booster in the first month of gestation.

The first pregnancy after treatment was complicated by severe preeclampsia, ending with a premature baby of 28 weeks of gestation, weighing 1,000 g. The next gestation, with the same treatment, was complicated by premature rupture of the membranes and ended with 32 weeks of gestation (1,250 g). At present both children are healthy and are attending school. The fifth gestation was unplanned and the medical treatment was started six weeks after conception. The fetus suffered intrauterine growth restriction and this finally caused intrauterine death at the 20^th^ week.

Antiphospholipid antibodies and lupus anticoagulant titers have been determined every two years and have been positive for the last ten years. To rule out an antiphospholipid syndrome secondary to systemic lupus erythematosus, the patient was evaluated and found negative for antinuclear and anti-DNA antibodies on several occasions.

## DISCUSSION

Anionic phospholipids are components of the inner faces of membranes, with a bilaminar conformation that is not antigenic. However, accessory proteins such as beta-2 glycoprotein I may facilitate and stabilize the transition to hexagonal antigenic forms. In general it is proposed that antiphospholipid antibodies are a mixture of antibodies against phospholipids and the beta-2 glycoprotein I complex or against phospholipids only.^7^

Controversy exists about the association of anticardiolipin antibodies with recurrent pregnancy loss. Many researchers have identified a relationship between them,^8,9^ but others refute such a link.^10^ Cardiolipin is restricted to the inner mitochondrial membrane,^3^ and several anionic phospholipids such as phosphatidylserine, phosphatidylinositol and phosphatidylethanolamine are components of plasma membranes. These antigens are more accessible to recognition by antibodies and can therefore be more relevant in recurrent pregnancy loss than cardiolipin.^11^

It has been proposed that a possible mechanism for recurrent pregnancy loss due to antiphospholipid antibodies could be thrombosis in placental and decidual vessels, and these antibodies might be able to inhibit the anticoagulant activity of antithrombin III, protein C, protein S and annexin V.^11,12^ For instance, annexin V is bound to phosphatidylserine and is removed from cell surfaces by antiphospholipid antibodies. This may be because these antibodies have a high affinity for phosphatidylserine or other phospholipids and the beta-2 glycoprotein I complex,^13^ which would increase prothrombin binding to endothelial and trophoblastic cell membranes.^7,14^

It has been demonstrated that antiphosphatidylserine monoclonal antibodies block the trophoblastic fusion and secretion of human chorionic gonadotrophin that are important for the continuation of pregnancy. In addition, while antiphosphatidylserine induced placental and intrauterine growth restriction in a murine model, this did not happen when anticardiolipin antibodies were given. These results suggest that obstetric complications associated with antiphospholipid syndrome could be caused by antiphospholipid antibody-induced trophoblastic dysfunction.^15^

Lymphocyte immunization used to be considered the conventional treatment for recurrent pregnancy loss associated with alloimmune failure. In 1994, the results of the International Collaborative Study and Metaanalysis on Allogenic Leukocyte Immunotherapy for recurrent pregnancy loss showed that this therapy was useful only in 8% to 10% of the patients. The use of this therapy is now very controversial and several authors recommend the withdrawal of this practice.^16^

Controlled clinical studies have evaluated the administration of heparin plus aspirin versus aspirin alone to patients with antiphospholipid syndrome and recurrent pregnancy loss, and have demonstrated an improvement in birth rate using the combined protocol (80% vs. 44%).^17^ Another study performed by Rai et al. led to similar results: 71% (32/ 45) in the group treated with heparin plus aspirin and 42% (19/45) in the group treated with aspirin alone (p < 0.01).^18^

Our research group has reported that preconceptional heparin plus aspirin therapy has an efficiency of 91% of live births (10/11), in patients with recurrent pregnancy loss associated with antiphospholipid syndrome and alloimmune failure, compared with 75% of live births (6/8) in patients treated with preconceptional immunization and postconceptional heparin.^19^ In the present case report, when the patient received preconceptional heparin and aspirin treatment, she successfully delivered a baby in two consecutive pregnancies.

Prednisone also has been used for preventing recurrent pregnancy loss in patients with antiphospholipid syndrome. In a randomized study, Silver et al.^20^ compared prednisone treatment plus low doses of aspirin (group #1) with aspirin treatment alone (group #2). The combined treatment resulted in a higher gestational success but the risk of preterm deliveries increased in this group (3/22 in group #1 and 8/12 in group #2, p = 0.003).

In spite of the controversy concerning all the treatments discussed in this report, and the fact that the pregnancy outcome was not completely successful due to the complications mentioned above, the final result was two kids at home. We consider that these combined therapies could be a good alternative in patients with antiphospholipid syndrome and alloimmune failure.
